# Predictors of Outcomes in Cerebellar Stroke: A Retrospective Cohort Study From the National Inpatient Sample Data

**DOI:** 10.7759/cureus.62025

**Published:** 2024-06-09

**Authors:** Ankita Prasad, Vinod Nookala, Riddhi Machchar, Jamarc R Simon, Lakshmi A Nakka, Twisha Vanamala, Sonia Mehta, Aishwarya Ramesh, Amber L Schilling, Christopher S Hollenbeak, Pramil Cheriyath

**Affiliations:** 1 Pediatrics, NewYork-Presbyterian Brooklyn Methodist Hospital, New York, USA; 2 Internal Medicine, Saint Clare’s Denville Hospital, Denville, USA; 3 Internal Medicine, Hackensack Meridian Ocean Medical Center, Brick, USA; 4 Anesthesiology, Yale University, New Haven, USA; 5 Surgery, Penn State Health Milton S. Hershey Medical Center, Hershey, USA; 6 Public Health, Penn State Health Milton S. Hershey Medical Center, Hershey, USA

**Keywords:** apr-drg scores, cerebellar stroke and cost and length of hospital stay, patient demographics, age, race, cerebellar stroke and its outcome and the type of medical insurance, mortality risk factors in cerebellar stroke, national inpatient sample (nis) and the healthcare cost and utilization project (hcup), retrospective cohort study, predictor of cerebellar stroke

## Abstract

Cerebellar strokes have high morbidity and mortality due to bleeding or edema, leading to increased pressure in the posterior fossa. This retrospective cohort study analyzed three outcomes following a cerebellar stroke: in-hospital mortality, length of hospital stay, and total hospitalization costs. It uses data from the National Inpatient Sample (NIS) and aims to identify the predictors of outcomes in cerebellar stroke patients, including 464,324 patients, 18 years of age and older, hospitalized between 2010 and 2015 in US hospitals with cerebellar strokes. In our study, for every decade age increased beyond 59 years, there was a significant increase in mortality; those aged 80+ years had 5.65 odds of mortality (95% CI: 5.32-6.00; P < 0.0001). Significant differences in patient characteristics were observed between patients who survived to discharge and those who did not, including older age (77.4 vs. 70.3 years; P < 0.0001), female sex (58% vs. 52%; P < 0.0001), and being transferred from another healthcare facility (17% vs. 10%; P < 0.0001). Patients admitted directly rather than through the emergency department were more likely to die (29% vs. 16%; P < 0.0001). The mortality rate was lower for blacks (OR: 0.75; P < 0.0001), Hispanics (OR: 0.91; P = 0.005), and Asians (OR: 0.89; P = 0.03), as compared to the white population, for females in comparison to males, and geographically, in all other areas (Midwest, South, and West) in contrast to the Northeast. Cerebellar stroke incidence and high mortality were seen in the traditional stroke belt. Mortality is also affected by the severity of the disease and increases with the Charlson Comorbidity Index (CCI), All Patient Refined Diagnosis Related Groups (APR-DRG) scores, and indirectly by place of receiving care, length of stay (LOS), cost of stay, type of insurance, and emergency department admissions. LOS increased with age, in males in the Northeast, and was less in whites compared to other races. Trend analysis showed a decrease in LOS and costs from 2010 to 2015. Increased costs were seen in non-whites, males, higher household income based on zip code, being covered under Medicaid, transfers, CCI ≥ 5, and discharges in the western US. Median household income based on the patient’s zip code was well-balanced between those who lived and those who died (P = 0.091). However, payers were not evenly distributed between the two groups (P < 0.0001 for the overall comparison). A higher proportion of discharges associated with in-hospital mortality were covered under Medicare (70% vs. 65% in the died vs. lived groups, respectively). Fewer discharges were associated with death if they were covered by commercial insurance or paid for out-of-pocket (15% vs. 19% for commercial insurance and 3% vs. 5% for out-of-pocket). In-hospital mortality was associated with a longer length of hospital stay (5.6 days vs. 4.5 days; P < 0.0001) and higher costs ($16,815 vs. $11,859; P < 0.0001). Variables that were significantly associated with lower total costs were older age, having commercial insurance, paying out-of-pocket or other payers, not being admitted through the emergency department, having a lower comorbidity index (CCI = 1-2), and being discharged from a hospital that was small- or medium-sized, located in the Midwest or South, and/or was non-teaching (rural or urban).

## Introduction

Stroke accounted for 41.1 fatalities per 100,000 people in 2021, making it the sixth most common cause of death in the US [1]. Cerebellar stroke accounts for 1-4% of strokes but causes much higher morbidity and mortality [2]. Standard clinical stroke scores frequently overlook the first presentation of vertigo, dizziness, nystagmus, headache, or vomiting in many patients with cerebellar stroke, which delays diagnosis and worsens outcomes [3]. To counteract the increasing intracranial pressure, cerebellar edema following a stroke may cause the cerebellar vermis to herniate upward. Cerebellar edema can block aqueducts and the fourth ventricle and cause hydrocephalus and, in severe cases, herniation of cerebellar tonsils in the foramen magnum, causing sudden death [3]. Numerous studies have reported that advanced age, low Glasgow Coma Scale (GCS) scores, and the development of hydrocephalus are substantial risk factors for adverse outcomes [3-6]. Both ischemic and hemorrhagic infarctions can result in cerebellar stroke, which is highly correlated with long-term hypertension and the use of anticoagulants or antiplatelets. Compared to other forms of stroke, cerebellar stroke is linked to much worse outcomes [6]. There is a paucity of demographic and socioeconomic data related to cerebellar stroke and its associated outcomes. Most of the studies attempt to identify clinical scores for predicting outcomes like stroke volume, GCS, comorbidities, and brain stem involvement [4-6]. Our research aims to determine the healthcare and socioeconomic factors that influence the outcomes of cerebellar stroke patients who are hospitalized and the impact of these predictors on cost and length of hospital stay in the US using nationally representative data.

## Materials and methods

Data source

Data for this study were collected from the National Inpatient Sample (NIS), a large administrative database maintained by the Healthcare Cost and Utilization Project (HCUP) and the Agency for Healthcare Research and Quality (AHRQ). The NIS is recognized as the largest publicly available all-payer inpatient healthcare database.

Cohort

In this retrospective study, we included patients in the NIS with hospital admission for cerebellar stroke between 2010 and 2015. Cerebellar stroke was identified using a principal International Classification of Diseases, Ninth Revision, Clinical Modification (ICD-9-CM) code of 434.91 (cerebral artery occlusion, unspecified, with cerebral infarction). After dropping patients under 18 years of age, as well as observations with missing demographics and outcomes, the final sample included 464,324 admissions with a diagnosis of cerebellar stroke.

Outcomes

Three outcomes following a cerebellar stroke were analyzed: in-hospital mortality, length of hospital stay, and total hospitalization costs. Mortality included only death prior to discharge; post-discharge mortality was not captured in the NIS. Length of hospital stay included all inpatient days from admission until discharge or death. Hospitalization costs reflect the hospital perspective and were estimated using the costs-to-charges ratio provided by the NIS. We adjusted for inflation over the time period represented in our sample using data from the medical care component of the consumer price index and adjusting all costs to 2018 US dollars.

Covariates

In multivariable analyses, we controlled for potential confounders, including demographics and health system characteristics. It was grouped into four categories: 18-59 years, 60-69 years, 70-79 years, and 80+ years. We also controlled for sex (male, female), race/ethnicity (white non-Hispanic, black non-Hispanic, Hispanic, Asian, other), and primary payer (Medicare, Medicaid, commercial, other). We controlled for socioeconomic status using the income quartile of the patient’s zip code as a proxy. Comorbidities were controlled using the Charlson Comorbidity Index (CCI). Several hospital characteristics were controlled, including hospital size (small, medium, large), teaching status, and urban/rural geography. We also controlled for whether the admission was transferred from another hospital. There were a few observations with missing values for covariates, which were dropped from the sample.

Statistical methods

The goal of the statistical analysis was to identify significant predictors of outcomes (mortality, length of stay (LOS), and costs) for patients admitted for cerebellar stroke. In descriptive analyses, patient and health system characteristics were compared between those who survived to discharge and those who did not using t-tests for continuous variables and chi-squared tests for binary and categorical variables. Mortality was modeled using logistic regression, controlling for other patient and health system characteristics. Linear models were fit to LOS and total costs, controlling for patient and health system characteristics. All analyses were performed using Stata version 15 software (StataCorp LLC, College Station, TX). Statistical significance was defined as P < 0.05.

## Results

**Figure 1:**
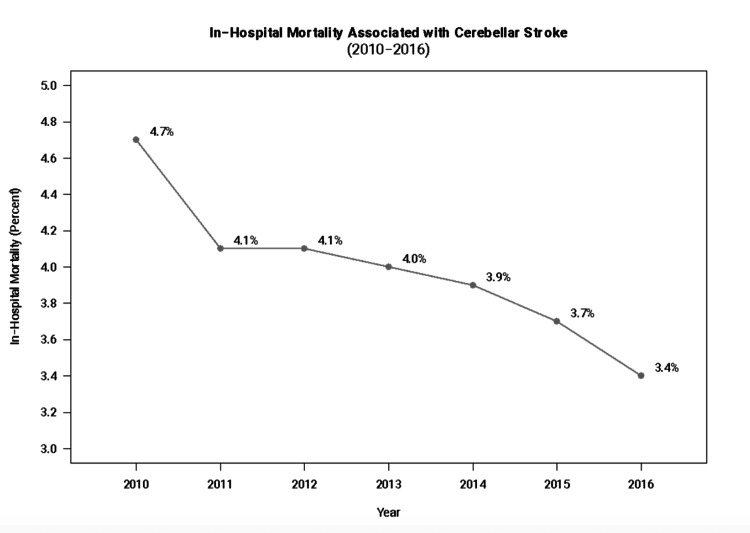
Trends over time for in-hospital mortality associated with cerebellar stroke (2010-2016).

Figure 1 presents the overall trend in mortality associated with cerebellar stroke between 2010 and 2016. As seen in Figure 1, the in-hospital mortality rate has steadily decreased from 4.7% in 2010 to 3.4% in 2016.

Table 1 provides patient demographics and clinical and hospital characteristics for the entire cerebellar stroke cohort (N = 462,324), stratified by in-hospital mortality. Significant differences in patient characteristics were observed between patients who survived to discharge and those who did not, including older age (77.4 vs. 70.3 years; P < 0.0001), female sex (58% vs. 52%; P < 0.0001), and being transferred from another healthcare facility (17% vs. 10%; P < 0.0001). Patients admitted directly rather than through the emergency department were more likely to die (29% vs. 16%; P < 0.0001), as were patients with a high comorbidity burden (CCI ≥ 5) and high All Patient Refined Diagnosis Related Groups (APR-DRG) severity risk score and APR-DRG mortality risk score. Hospital characteristics such as number of beds, region, and location-teaching status also differed significantly between patients who survived and those who died in the hospital (P < 0.0001 for all comparisons). The distribution of median household income based on the patient’s zip code was well-balanced between those who lived and those who died (P = 0.091). However, payers were not evenly distributed between the two groups (P < 0.0001 for the overall comparison). A higher proportion of discharges associated with in-hospital mortality were covered under Medicare (70% vs. 65% in the died vs. lived groups, respectively). Fewer discharges were associated with death if they were covered by commercial insurance or paid for out-of-pocket (15% vs. 19% for commercial insurance and 3% vs. 5% for out-of-pocket).

**Table 1:** Demographic characteristics and outcomes for all admissions associated with a primary diagnosis code of cerebellar stroke stratified by in-hospital mortality. APR-DRG, All Patient Refined Diagnosis Related Groups; LOS, length of stay.

	Lived	Died	
Variable	(N = 444,009)	(N = 18,315)	P-value
Age (mean, years)	70.3	77.4	<0.0001
18-59	24%	11%	
60-69	22%	14%	
70-79	23%	21%	
80+	31%	54%	
Race/ethnicity			<0.0001
White	64%	70%	
Black	17%	11%	
Hispanic	7%	6%	
Asian	2%	2%	
Other	9%	10%	
Sex			<0.0001
Male	48%	42%	
Female	52%	58%	
Median household income based on patient's zip code			0.091
0-25th percentile	32%	31%	
26th-50th percentile	26%	27%	
51st-75th percentile	23%	23%	
76th-100th percentile	19%	19%	
Payer			<0.0001
Medicare	65%	70%	
Medicaid	8%	5%	
Commercial	19%	15%	
Self-pay	5%	3%	
Other	3%	6%	
Transfer status			
Transferred	10%	17%	<0.0001
Not transferred	90%	83%	
Admitted through the emergency department			<0.0001
Yes	84%	71%	
No	16%	29%	
Charlson Comorbidity Index			<0.0001
1-2	33%	27%	
3-4	38%	33%	
≥5	29%	40%	
APR-DRG severity			<0.0001
1	13%	3%	
2	53%	14%	
3	29%	35%	
4	6%	48%	
APR-DRG mortality risk			<0.0001
1	34%	2%	
2	43%	18%	
3	18%	23%	
4	5%	57%	
Hospital bed size			<0.0001
Small	14%	13%	
Medium	27%	26%	
Large	59%	61%	
Hospital region			<0.0001
Northeast	16%	18%	
Midwest	23%	22%	
South	44%	43%	
West	17%	17%	
Location & teaching status			<0.0001
Rural	11%	14%	
Urban (non-teaching)	36%	32%	
Urban (teaching)	53%	54%	
LOS (mean, days)	4.5	5.6	<0.0001
0-2 days	34%	35%	
3-4 days	34%	23%	
5-7 days	19%	20%	
≥8 days	13%	22%	
Total cost (mean, 2018 US dollars)	$11,859	$16,815	<0.0001
$7,000 and less	33%	34%	
$7,000-$9,999	26%	13%	
$10,000-$14,999	21%	16%	
≥$15,000	20%	36%	

Table 2 presents the logistic regression model for the odds of death among discharges associated with cerebellar stroke. Each age band above 18-59 years was associated with significantly higher odds of death following cerebellar stroke. Most impressive was that age ≥80 years had an odd of in-hospital mortality over five times that of the youngest age group (OR = 5.65; P < 0.0001). Being Afro-American, Hispanic, or Asian was associated with fewer deaths compared to being Caucasian (OR = 0.75, OR = 0.91, and OR = 0.89, respectively; P < 0.0001, P = 0.005, and P = 0.03, respectively). As suggested in the univariate analysis, being male was also associated with lesser risk (OR = 0.94; P < 0.0001). Discharges covered under Medicaid, commercial insurance, self-payment, or other payment means were all associated with significantly higher odds of mortality compared to those covered under Medicare (OR = 1.53, 1.62, 1.99, and 3.50, respectively; P < 0.0001 for all variables), which is slightly opposite to what the univariate analysis showed. However, consistent with the univariate analysis, being transferred from another healthcare facility was associated with significantly higher odds of death (OR = 1.36; P < 0.0001).

**Table 2:** Logistic regression model for mortality. Area under the receiver operating curve (AROC) = 0.69; N = 462,324.

		95% CI		
Variable	Odds ratio	Lower	Upper	P-value
Age (years)				
18-59	Reference			
60-69	1.63	1.5312	1.7323	<0.0001
70-79	2.88	2.707	3.0743	<0.0001
80+	5.65	5.3191	5.9993	<0.0001
Race/ethnicity				
White	Reference			
Black	0.75	0.7142	0.7892	<0.0001
Hispanic	0.91	0.8557	0.9724	0.005
Asian	0.89	0.8026	0.989	0.03
Other	1.04	0.9842	1.0915	0.175
Sex				
Male	0.94	0.9089	0.9669	<0.0001
Female	Reference			
Median household income based on patient's zip code				
0-25th percentile	Reference			
26th-50th percentile	0.96	0.925	1.0029	0.069
51st-75th percentile	0.93	0.8936	0.9748	0.002
76th-100th percentile	0.94	0.9012	0.9908	0.019
Payer				
Medicare	Reference			
Medicaid	1.53	1.4178	1.6527	<0.0001
Commercial	1.62	1.5466	1.701	<0.0001
Self-pay	1.99	1.8185	2.1723	<0.0001
Other	3.5	3.2611	3.7489	<0.0001
Transfer status				
Transferred	1.36	1.2964	1.4197	<0.0001
Not transferred	Reference			
Admitted through the emergency department				
Yes	Reference			
No	1.93	1.856	2.0041	<0.0001
Charlson Comorbidity Index				
1-2	0.87	0.841	0.9086	<0.0001
3-4	Reference			
≥5	1.49	1.443	1.5487	<0.0001
Hospital bed size				
Small	0.81	0.7751	0.8498	<0.0001
Medium	0.89	0.8591	0.9224	<0.0001
Large	Reference			
Hospital region				
Northeast	Reference			
Midwest	0.68	0.6485	0.7173	<0.0001
South	0.9	0.8582	0.9374	<0.0001
West	0.83	0.7897	0.8773	<0.0001
Location & teaching status				
Rural	0.97	0.9266	1.0211	0.264
Urban (non-teaching)	0.88	0.8548	0.9157	<0.0001
Urban (teaching)	Reference			

**Figure 2:**
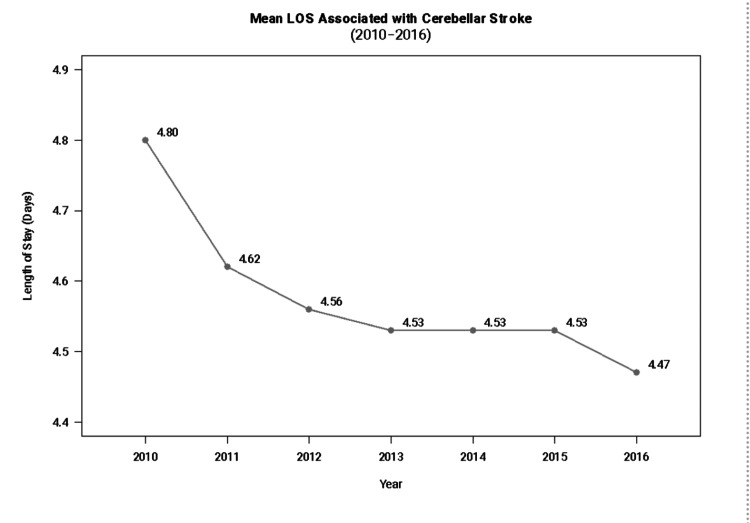
Trends over time for length of stay (LOS) associated with cerebellar stroke (2010-2016).

Trends in the average length of hospital stay are presented in Figure 2, which shows that mean LOS among patients admitted for cerebellar stroke has steadily declined since 2010. Mean LOS fell slightly from 4.80 days in 2010 to 4.47 days in 2016.

The linear regression model for hospital LOS is provided in Table 3. A patient discharged with a diagnosis of cerebellar stroke that possessed characteristics of each reference group was associated with a length of stay of 4.68 days. Characteristics that significantly extended LOS were older age, being non-white, being covered under Medicaid or being self-paying, being transferred from another healthcare facility, not being admitted through the emergency department, and/or having a higher comorbidity index (CCI ≥ 5). Variables that significantly shortened LOS included higher income quartile, being covered under commercial insurance, having a lower comorbidity index (CCI = 1-2), and being discharged from a hospital that was small- or medium-sized, located outside of the Northeast, and/or was non-teaching (rural or urban).

**Table 3:** Linear regression model for length of stay (LOS). R2 = 0.0415; N = 462,324.

		95% CI		
Variable	Coefficient	Lower	Upper	P-value
Age (years)				
18-59	Reference			
60-69	0.21	0.1563	0.2561	<0.0001
70-79	0.32	0.2663	0.3788	<0.0001
80+	0.57	0.5195	0.6299	<0.0001
Race/ethnicity				
White	Reference			
Black	0.89	0.8403	0.931	<0.0001
Hispanic	0.49	0.4241	0.5505	<0.0001
Asian	0.66	0.5589	0.7668	<0.0001
Other	0.27	0.2161	0.3288	<0.0001
Sex				
Male	0.05	0.0196	0.0835	0.002
Female	Reference			
Median household income based on patient's zip code				
0-25th percentile	Reference			
26th-50th percentile	-0.14	-0.1776	-0.0932	<0.0001
51st-75th percentile	-0.19	-0.2308	-0.1409	<0.0001
76th-100th percentile	-0.17	-0.2199	-0.1208	<0.0001
Payer				
Medicare	Reference			
Medicaid	1.8	1.733	1.8678	<0.0001
Commercial	-0.22	-0.2702	-0.1728	<0.0001
Self-pay	0.43	0.3467	0.5082	<0.0001
Other	0.2	0.1066	0.2974	<0.0001
Transfer status				
Transferred	0.76	0.7055	0.821	<0.0001
Not transferred	Reference			
Admitted through the emergency department				
Yes	Reference			
No	0.41	0.3633	0.4573	<0.0001
Charlson Comorbidity Index				
1-2	-0.66	-0.6944	-0.6195	<0.0001
3-4	Reference			
≥5	0.98	0.9441	1.0211	<0.0001
Hospital bed size				
Small	-0.64	-0.6892	-0.5954	<0.0001
Medium	-0.34	-0.3721	-0.2988	<0.0001
Large	Reference			
Hospital region				
Northeast	Reference			
Midwest	-0.95	-1.006	-0.8997	<0.0001
South	-0.33	-0.3788	-0.2838	<0.0001
West	-0.79	-0.8469	-0.7345	<0.0001
Location & teaching status				
Rural	-0.74	-0.7918	-0.6851	<0.0001
Urban (non-teaching)	-0.38	-0.4168	-0.3466	<0.0001
Urban (teaching)	Reference			
Intercept	4.68	4.6032	4.7581	<0.0001

**Figure 3:**
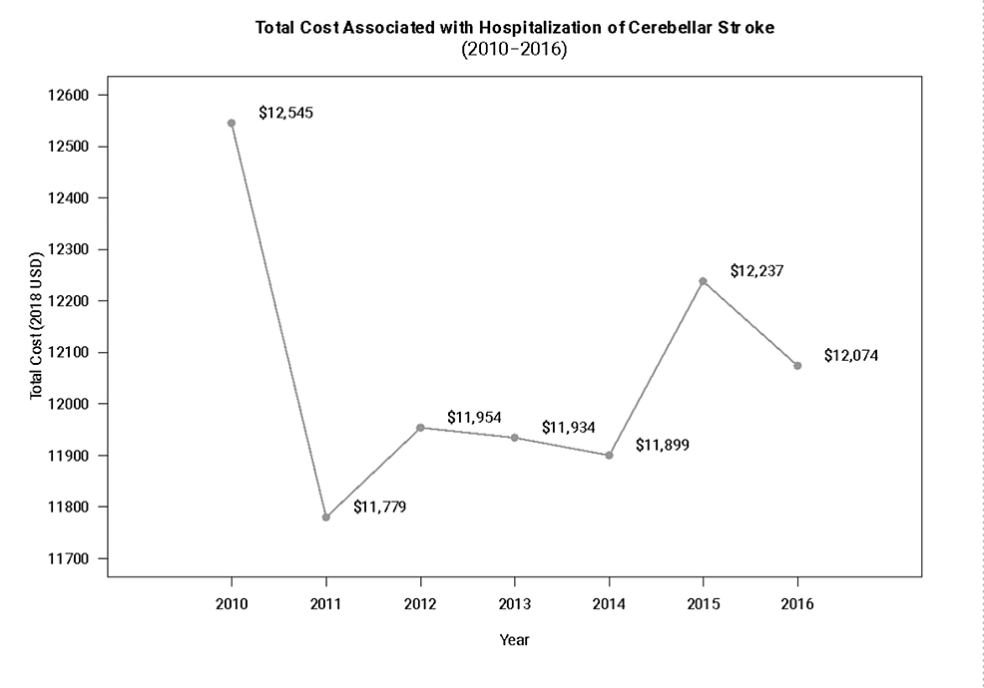
Trends over time for mean total cost associated with cerebellar stroke (2010-2016).

Figure 3 presents the trend over time for total costs associated with cerebellar stroke discharges. Overall, costs have decreased by approximately $470 between 2010 and 2016.

Results from the multivariable model of total hospitalization costs are presented in Table 4. The intercept term suggests that a patient discharged with a diagnosis of cerebellar stroke that possessed characteristics of each reference group had expected costs of $13,995 (2018 US dollars) per hospitalization. Characteristics that significantly increased costs included being non-white, male sex, having a higher household income based on zip code, being covered under Medicaid, being transferred from another healthcare facility, having a higher comorbidity index (CCI ≥ 5), and being discharged from a hospital in the western region of the US. Variables that were significantly associated with lower total costs were older age, having commercial insurance, paying out-of-pocket or other payers, not being admitted through the emergency department, having a lower comorbidity index (CCI = 1-2), and being discharged from a hospital that was small- or medium-sized, located in the Midwest or South, and/or was non-teaching (rural or urban).

**Table 4:** Linear regression model for total costs (in 2018 US dollars). R2 = 0.0448; N = 462,324.

		95% CI		
Variable	Coefficient	Lower	Upper	P-value
Age (years)				
18-59	Reference			
60-69	-$486	-$608	-$364	<0.0001
70-79	-$650	-$787	-$512	<0.0001
80+	-$1,394	-$1,529	-$1,259	<0.0001
Race/ethnicity				
White	Reference			
Black	$1,441	$1,330	$1,551	<0.0001
Hispanic	$1,634	$1,480	$1,788	<0.0001
Asian	$2,090	$1,836	$2,343	<0.0001
Other	$960	$823	$1,098	<0.0001
Sex				
Male	$357	$279	$435	<0.0001
Female	Reference			
Median household income based on patient's zip code				
0-25th percentile	Reference			
26th-50th percentile	$74	-$29	$177	0.161
51st-75th percentile	$429	$319	$539	<0.0001
76th-100th percentile	$1,331	$1,210	$1,452	<0.0001
Payer				
Medicare	Reference			
Medicaid	$2,700	$2,535	$2,864	<0.0001
Commercial	-$196	-$315	-$77	0.001
Self-pay	-$52	-$249	$145	0.603
Other	-$429	-$661	-$196	<0.0001
Transfer status				
Transferred	$1,717	$1,576	$1,858	<0.0001
Not transferred	Reference			
Admitted through the emergency department				
Yes	Reference			
No	-$693	-$808	-$579	<0.0001
Charlson Comorbidity Index				
1-2	-$1,327	-$1,418	-$1,235	<0.0001
3-4	Reference			
≥5	$2,097	$2,003	$2,191	<0.0001
Hospital bed size				
Small	-$1,438	-$1,552	-$1,324	<0.0001
Medium	-$1,064	-$1,153	-$975	<0.0001
Large	Reference			
Hospital region				
Northeast	Reference			
Midwest	-$1,916	-$2,045	-$1,786	<0.0001
South	-$2,320	-$2,436	-$2,204	<0.0001
West	$1,077	$940	$1,215	<0.0001
Location & teaching status				
Rural	-$2,237	-$2,367	-$2,107	<0.0001
Urban (non-teaching)	-$1,846	-$1,931	-$1,760	<0.0001
Urban (teaching)	Reference			
Intercept	$13,995	$13,806	$14,184	<0.0001

## Discussion

This observational cohort study examined predictors of cerebellar stroke outcomes, LOS, and the cost of hospitalization. Trend analysis of our data showed a steady decrease in overall mortality, LOS, and total costs from 2010 to 2016. We found that several demographic and health system characteristics were associated with in-hospital mortality, length of hospital stay, and total hospitalization cost for patients admitted with a principal diagnosis of cerebellar stroke. We do recognize that socioeconomic factors play a very important role in cerebellar stroke outcomes, but many of the socioeconomic factors are linked in complex ways. Traditionally, we associate demographic factors like being white or female, having better access to care, and not being in the traditional stroke belt with better stroke numbers and outcomes, but these demographic factors are also associated with increased lifespan, and age was the strongest predictor of mortality in our study. In 2019, the death rate from all cerebrovascular diseases was 977 per 100,000 people aged 85 years and older, and just 30.5 per 100,000 among those aged 55 to 64 years [7]. Even though cerebellar strokes cause 1-4% of all strokes [2], the outcome is much poorer, especially in the elderly. Some other studies have also found that advanced age can predict an unfavorable outcome [4-6]. When evaluating the patient demographics and their relationship with cerebellar stroke outcomes, we found that the mean age of people who died was 77.4 years as compared to 70.3 years for those who survived (P < 0.0001), and patients in the 80+ age group had significantly higher in-hospital mortality, and their odds of in-hospital mortality were more than five times higher as compared to the youngest patients. In our study, males had a slightly lower mortality risk than females. Of patients who died, 58% were women, compared to 42% of men. This difference was statistically significant. For some other studies, gender was a significant outcome predictor [8-10]. Cerebellar stroke affects a greater number of women because of their increased life span and the fact that stroke event rates increase substantially in the oldest age groups [9-11]. Moreover, stroke-related outcomes, including disability and quality of life (QOL), are consistently poorer in women than in men [11]. The fact that older women are much more likely to live alone and be socially isolated increases the societal impact of poor stroke outcomes in women. Considering the exact reasons for cerebellar stroke as well as other types of strokes, we can explain this difference in mortality in our study. White patients, which comprised 64% of the study population, had a statistically significant higher rate of death following cerebellar stroke and higher odds of dying as compared to the other racial and ethnic groups. This contrasts with previous studies, which have generally suggested a higher incidence of stroke at younger ages in black patients [9,12,13]. Age differences between racial and ethnic groups are related to cerebellar stroke incidence and outcomes and likely explain some of this observation [9].

We also studied the outcomes based on household income and grouped patients into income percentiles based on zip codes. We considered zip codes as a proxy for household income and better access to health care, based on earlier studies [14]. However, the distribution of median household income based on the patient’s zip code was well-balanced between those who lived and those who died (P = 0.091). However, in logistic regression, for mortality, we found marginal but significantly lower odds of mortality in the zip codes with an income percentile greater than 50%, so better access to health care does influence outcomes. When evaluating the relationship between cerebellar stroke and its outcome and the type of medical insurance the person had, we found a significant difference between patients with Medicare and other payers, i.e., 70% vs. 65% in the died vs. lived groups, respectively. The fact that Medicare patients are typically elderly likely explains this. It also reiterates the previous data seen for stroke outcomes based on medical insurance types [15,16]. There are many reasons for this, which can be a lack of primary prevention, poor management of comorbidities, and delay in seeking treatment [16]. However, in contrast to the significantly higher in-hospital mortality in univariate analysis, the logistic regression showed discharges covered under Medicaid, commercial insurance, self-payment, or other payment means were all associated with significantly higher odds of mortality compared to those covered under Medicare. In a study by Fargen et al., those without insurance and Medicaid beneficiaries had 1.5 times higher odds of experiencing a poor outcome and 1.2 times higher odds of dying during their stroke hospitalizations and patients with private insurance had a significantly better outcome compared to Medicaid and no insurance [17].

Patients who are transferred out to other centers are usually more critically sick and interhospital transfers are for escalation of care. However, the transfer causing delay in getting timely intervention in stroke patients has been shown in other studies to affect the outcomes of stroke [18]. This is reflected in significantly higher mortality in transfer cases in our study. Patients not admitted through the emergency department (ED) had significantly higher mortality in our study. This difference signifies a delay in care for patients not admitted through ED accounting for higher mortality. Al Safatli et al. used the GCS as a predictor of mortality in their study and found higher odds of mortality with low GCS; they found the best cutoff for GCS of 10 at admission [4]. For our study, we have used the CCI, which summarizes the overall comorbidity burden as a weighted index to predict the risk of death within one year of hospitalization for patients with specific comorbid conditions. It is one of the more common criteria used to measure the comorbidity of individual episodes in hospitalized patients. Patients with a CCI of 5 or more are at a significantly increased risk of in-hospital mortality from cerebellar stroke, as they are sicker than patients with scores of 1-4. The APR-DRG is a classification system that classifies patients according to their reason for admission, illness severity, and mortality risk [6]. The APR-DRG scores are used by Medicare, calculated from discharge billing codes, and based on primary and secondary discharge diagnosis, age, and pre-existing medical conditions. APR-DRG ranks the risk of mortality as low, medium, high, and extreme based on scores. This explains the significantly increased risk of in-hospital mortality as the APR-DRG severity score increases. Patients admitted to smaller hospitals with fewer beds and rural hospitals had significantly lower mortality rates than those admitted to hospitals with larger numbers of beds. Those in urban non-teaching hospitals had significantly lower mortality rates than those in urban teaching hospitals, as they had the most sick patients. Patients who are less ill have a low risk of mortality and can be conveniently managed in even smaller rural hospitals. The southern US is known as the "stroke belt" and there are significant geographic variations in the stroke death rate among the US states. The southeastern United States features the highest concentrations of people living with cardiovascular and stroke risk factors like obesity, high blood pressure, diabetes, and smoking [19,20], which are known risk factors for stroke. Our study shows geographic variation in mortality and LOS very different from this and patients from the Midwest, South, and West had significantly lesser mortality as compared to patients from the Northeast.

We observed an extended LOS associated with older age, being non-white, being covered under Medicaid or self-paying, being transferred from another healthcare facility, not being admitted through the emergency department, and/or having a higher comorbidity index (CCI ≥ 5). Variables that significantly shortened LOS included higher income quartile, being covered under commercial insurance, having a lower comorbidity index (CCI = 1-2), and being discharged from a hospital that was small- or medium-sized, located outside of the Northeast, and/or was non-teaching (rural or urban). We also found significantly increased costs associated with being non-white, male sex, having a higher household income based on zip code, being covered under Medicaid, being transferred in from another healthcare facility, having a higher comorbidity index (CCI ≥ 5), and being discharged from a hospital in the western region of the US. Variables that were significantly associated with lower total costs were older age, having commercial insurance, paying out-of-pocket, or other payers, not being admitted through the emergency department, having a lower comorbidity index (CCI = 1-2), and being discharged from a hospital that was small- or medium-sized, located in the Midwest or South, and/or was non-teaching (rural or urban).

As for the clinical markers, various studies have shown the size, location, and type of cerebellar stroke (ischemic or hemorrhagic), along with the GCS at presentation and the presence or absence of hydrocephalus and drainage requirements, have a bearing on its clinical outcome [4,5,21,22]. This correlates with higher mortality and LOS for patients with higher CCI and APR-DRG scores. In a study by Appelros et al., age, sex, living alone, and dementia were major risk factors for severe stroke and poor outcomes [23].

We recognize this study has several limitations, many of which are common to observational studies using large administrative datasets. While the data are nationally representative, there are still concerns about the ability to generalize to specific populations. While we were able to control for many patient and health system characteristics, there are other potential confounders that were noted in the dataset and for which we could not control. Finally, because our models do not support causal inference, we are limited to identifying associations between outcomes and covariates.

## Conclusions

Cerebellar stroke is associated with increased mortality. Through this study, we tried to understand the socioeconomic and demographic factors that influence mortality, LOS, and hospitalization costs in cerebellar stroke patients. We derived our data from the NIS from patients older than 18 years admitted from 2010 to 2015 with cerebellar stroke. Trend analysis of our data showed a steady decrease in overall mortality from cerebellar stroke, LOS, and total costs from 2010 to 2016. We identified age, race, sex, and region of the country as important predictors of mortality. As age increased, mortality and LOS increased substantially. White Americans had the highest incidence and mortality rate, compared to Hispanics, Asians, and black Americans. We still need to compare the effect of age versus other socioeconomic determinants to find the exact relationship. Males had reduced odds of mortality. Mortality is also affected by the severity of the disease, as shown directly by its relationship to the CCI and APR-DRG scores, as well as indirectly by its association with the place of receiving care, LOS, cost of stay, and emergency department admissions. There was a strong regional correlation, with higher mortality seen in the Northeast of the US. An extended LOS is associated with older age, being non-white, being covered under Medicaid or being self-paying, being transferred in from another healthcare facility, not being admitted through the emergency department, and/or having a higher comorbidity index (CCI ≥ 5). Variables that significantly shortened LOS included a higher income quartile, being covered under commercial insurance, having a lower comorbidity index (CCI = 1-2), and being discharged from a hospital that was small- or medium-sized, located outside of the Northeast, and/or was non-teaching (rural or urban). We also found significantly increased costs associated with being non-white, male, having a higher household income based on zip code, being covered under Medicaid, being transferred in from another healthcare facility, having a higher comorbidity index (CCI ≥ 5), and being discharged from a hospital in the western region of the US. Variables that were significantly associated with lower total costs were older age, having commercial insurance, paying out-of-pocket, or other payers, not being admitted through the emergency department, having a lower comorbidity index (CCI = 1-2), and being discharged from a hospital that was small- or medium-sized, located in the Midwest or South, and/or was non-teaching (rural or urban).
